# TrkC promotes colorectal cancer growth and metastasis

**DOI:** 10.18632/oncotarget.17289

**Published:** 2017-04-20

**Authors:** Min Soo Kim, Kwang Wook Suh, Suntaek Hong, Wook Jin

**Affiliations:** ^1^ Laboratory of Molecular Disease and Cell Regulation, Department of Biochemistry, School of Medicine, Gachon University, Incheon 406-840, Korea; ^2^ Department of Surgery, Ajou University School of Medicine, Yeongto-gu, Suwon 443-380, Korea; ^3^ Laboratory of Cancer Cell Biology, Department of Biochemistry, School of Medicine, Gachon University, Incheon 406-840, Korea; ^4^ Gachon Medical Research Institute, Gil Medical Center, Incheon 405-760, Korea

**Keywords:** TrkC, colorectal cancer, tumorigenicity, metastasis, EMT program

## Abstract

The current work reveals that TrkC receptor is crucial to many aspects of tumorigenicity and metastasis of cancer. However, with only a few exceptions, such as colorectal cancer (CRC), where suppressing tumorigenic and metastatic ability via expression of TrkC as tumor suppressor have been proposed. These diverse lines of evidence led us to investigate whether TrkC is involved in CRC progression. By using mouse models and molecular biology analyses, we demonstrate that TrkC acts as an activator in tumorigenicity and metastasis of colorectal cancer. In this study, TrkC was frequently overexpressed in CRC cells, patients’ tumor samples and an azoxymethane/dextran sulphate sodium-induced mouse model of colitis-associated CRCs. TrkC expression was associated with a high-grade CRC phenotype, leading to significantly poorer survival. Also, TrkC expression promoted the acquisition of motility and invasiveness in CRC. Moreover, TrkC increased the ability to form tumor spheroids, a property associated with cancer stem cells. Importantly, knockdown of TrkC in malignant mouse or human CRC cells inhibited tumor growth and metastasis in a mouse xenograft model. Furthermore, TrkC enhanced metastatic potential and induced proliferation by aberrant gain of AKT activation and suppression of transforming growth factor (TGF)-β signalling. Interestingly, TrkC not only modulated the actions of TGF-β type II receptor, but also attenuated expression of this receptor. These findings reveal an unexpected physiological role of TrkC in the pathogenesis of CRC. Therefore, TrkC is a potential target for designing effective therapeutic strategies for CRC development.

## INTRODUCTION

In addition to their function of TrkC receptor in neuronal survival, accumulating evidence also implicates TrkC in cancer. TrkC, induces tumor invasiveness and chemotaxis of malignant cells [1]. TrkC is also important in the regulation of angiogenesis [2], induction of tumor growth [3], prevention of apoptosis [4] and promotion of metastasis [5]. Moreover, TrkC is activated by fusion with multimerising proteins. Fusion of TrkC with ETV6 generates the ETV6-NTRK3 chimeric tyrosine kinase, which is suggested to contribute to oncogenesis via dysregulation of TrkC downstream signal transduction pathways [6]. In addition to activation of TrkC by fusion with multimerising proteins, TrkC has a high capacity for ligand-independent activation, presumably via spontaneous interactions. Thus, high levels of TrkC expression elicit autophosphorylation in the absence of neurotrophin-3 (NT-3) [7]. Furthermore, TrkC appears to be activated by overexpression of the full-length protein in a number of human tumors such as neuroblastoma, paediatric brain tumors, breast cancer, liver cancer, leukaemia, leiomyosarcoma, melanoma, pancreatic and prostate carcinoma, and basal cell and cutaneous squamous cell carcinoma [3–5, 7–10].

Although TrkC plays an important role in the initiation and progression of many tumors, several recent studies proposed that TrkC is a tumor suppressor as a dependence receptor in CRC. As the original meaning, dependence receptors are dependent on ligand availability to trigger two completely opposite signalling pathways. In the presence of ligand, these receptors activate classic signalling pathways implicated in cell survival, migration and tumor progression. However, these receptors act as tumor suppressors to promote cell death in the absence of ligand [11]. Current studies report that NT-3 and TrkC expression was significantly elevated in normal colon tissues than in primary CRC tissues and CRC cells but NT-3 treatment of colon cancer cells ectopically expressing TrkC inhibits the tumor suppressor activity of TrkC. Also, Reconstitution of TrkC induces apoptosis in colorectal cancers when NT-3 is absent [12, 13]. However, we recently reported that TrkC blocks bone morphogenetic protein 2 (BMP2)-mediated tumor suppressor activity in colon cancer [14]. Therefore, these results raise the question of whether TrkC acts as an oncogenic protein or tumor suppressor in CRC progression and whether NT-3 suppresses TrkC-mediated apoptosis in CRC cells, despite NT-3 and TrkC being reportedly upregulated in normal colon relative to CRC tissues and cells. These diverse lines of evidence led us to investigate whether TrkC is involved in CRC progression. In this report, we show that TrkC can lead to deregulated cell growth, alters cellular behaviour and function, and enhanced metastasis of CRC.

## RESULTS

### TrkC expression is associated with CRC pathogenesis

**Figure 1:**
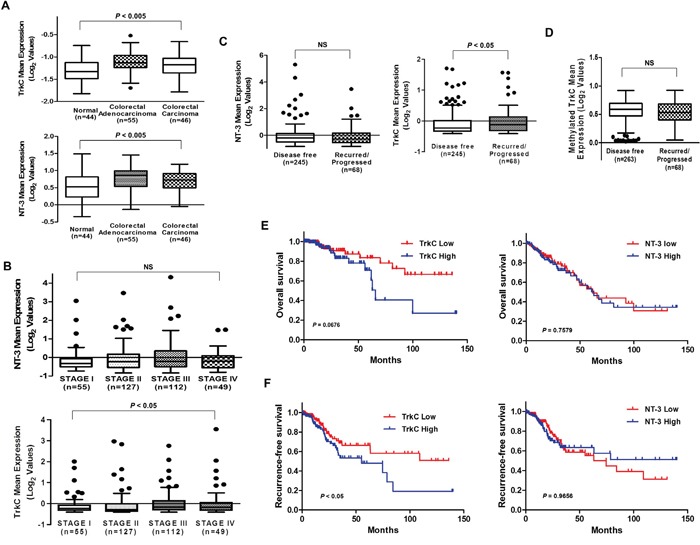
Correlation of TrkC with CRC pathogenesis and patient survival **(A)** Box-and-whisker (Tukey) plots of the mean expression of TrkC and NT-3 in CRC patients. TrkC and NT-3 levels were extracted from the Skrzypczak microarray dataset (GSE20916) and averaged in each tumor. Points below and above the whiskers are drawn as individual dots. *P* < 0.05 was considered to indicate significance in ANOVA. **(B)** TrkC expression is correlated with the stages of CRC. Mean expression of TrkC and NT-3, obtained through RNA-sequence analysis of 629 CRC patients in the TCGA dataset, were plotted as box plots according to the tumor stages. TrkC and NT-3 levels were extracted from the dataset and averaged in each tumor. Points below and above the whiskers are drawn as individual dots. *P* < 0.05 was considered to indicate significance in ANOVA. NS, not significant. **(C)** TrkC expression is correlated with recurrence in CRC patients, but NT-3 expression is not. Mean expression of TrkC and NT-3, obtained by RNA-sequence analysis of 629 CRC patients in the TCGA dataset, was plotted as box plots according to the disease-free status of CRC patients. TrkC and NT-3 levels were extracted from the dataset and averaged in each tumor. Points below and above the whiskers are drawn as individual dots. The Student's t-test was performed to assess statistical significance (*P < 0.05). **(D)** Mean methylated TrkC expression, obtained by analysis of the Infinium Human Methylation 450 BeadChip array (HM450) of 331 CRC patients in the TCGA dataset, was plotted as box plots. TrkC levels were extracted from the dataset and averaged in each tumor. Points below and above the whiskers are drawn as individual dots. *P* < 0.05 was determined by the Student's t-test. NS, not significant. **(E, F)** In total, 629 CRC patients from the TCGA dataset were divided into high and low TrkC or NT-3 expressers, and overall **(E)** and recurrence-free **(F)** survival were compared. *P* values correspond to the log-rank test comparing the survival curves.

To investigate the possible correlation between TrkC and CRC progression, we initially performed *in silico* analysis of TrkC expression using a large clinical study from Oncomine. Interestingly, TrkC expression was strongly correlated with the signature derived from CRC patients through analysis of TrkC and NT-3 expression using several publicly available datasets and patient clinical data. TrkC and NT-3 expression in GSE20916 [15] was markedly upregulated in CRC tissues of patients relative to normal tissue samples (Figure 1A). In addition, TrkC expression in the GSE28722 [16] and TCGA [17, 18] datasets was significantly upregulated in other stages (III, IV) than in stage I of CRC; however, NT-3 expression did not significantly differ from between CRC stages (Figure 1B and Supplementary Figure 1A). Moreover, NT-3/TrkC expression did not significantly differ from CRC stages (Supplementary Figure 1B). Furthermore, we found an indirect correlation between NT-3 expression and TrkC expression through correlation analysis in the GSE20916, GSE28722 and TCGA datasets (Supplementary Figure 1C). Our findings are in contrast to a previous study, which demonstrated that TrkC and NT-3 expression was significantly lower in CRC than in normal colon via tumor-associated promoter methylation and TrkC expression was significantly correlated with NT-3 expression [12, 13].

Based on these observations, we next examined whether TrkC expression was associated with CRC recurrence. Interestingly, analysis of 313 CRC patients in the TCGA dataset showed that TrkC expression was elevated in patients with recurred/progressed CRC relative to non-recurred CRC patients. However, NT-3 expression did not significantly differ between recurred/progressed and non-recurred CRC patients (Figure 1C), suggesting that TrkC expression is involved in CRC progression and CRC recurrence. The level of methylated TrkC did not significantly differ between non-recurred and recurred CRC patients based on analysis of the Infinium Human Methylation 450 BeadChip array (HM450) in the TCGA dataset (Figure 1D). Our results suggest that TrkC may contribute to higher invasive recurrence risk of CRC through overexpression of TrkC mRNA, consistent with previous report that 14 of 17 patients with CRC were found coexpression of TrkC/NT-3 by immunohistochemical analysis and TrkC was related with metastasis [19].

To test whether TrkC expression was correlated with the survival status of CRC patients, we conducted Kaplan-Meier survival analysis using patient clinical data of the TCGA and GSE28722 datasets. By analysing the association of TrkC expression with the survival status of CRC patients, we further confirmed that gain of TrkC expression correlated with poorer overall survival and recurrence-free survival. However, survival status did not significantly differ between patients with tumors expressing high NT-3 levels and tumors expressing low NT-3 levels (Figure 1E, 1F and Supplementary Figure 1D).

TCGA integrative analysis found new molecular signatures associated with tumor aggressiveness on the basis of tumor stage, lymph node status, distant metastasis and vascular invasion at the time of surgery. There were 11 molecular markers associated with tumor aggressiveness and 19 molecular markers associated with less-aggressive tumor [18]. Surprisingly, TrkC expression was significantly correlated with all the new molecular markers of aggressive CRCs. High TrkC expression markedly induced 10 of 11 markers of more aggressive CRCs including SCN5A, a reported regulator of colon cancer invasion (Supplementary Figure 2A). Moreover, expression of 8 of 19 markers of less aggressive CRCs was significantly reduced when TrkC expression was high (Supplementary Figure 2B), but expression of 11 of these 19 markers did not differ according to whether TrkC expression was high or low (data not shown). This suggests that TrkC expression might plays a crucial role in the initiation, progression and metastasis of CRCs but not tumor suppressor.

### TrkC expression is increased in human CRC patient samples and an azoxymethane (AOM)/dextran sulphate sodium (DSS)-induced mouse model of colitis-associated CRCs

**Figure 2:**
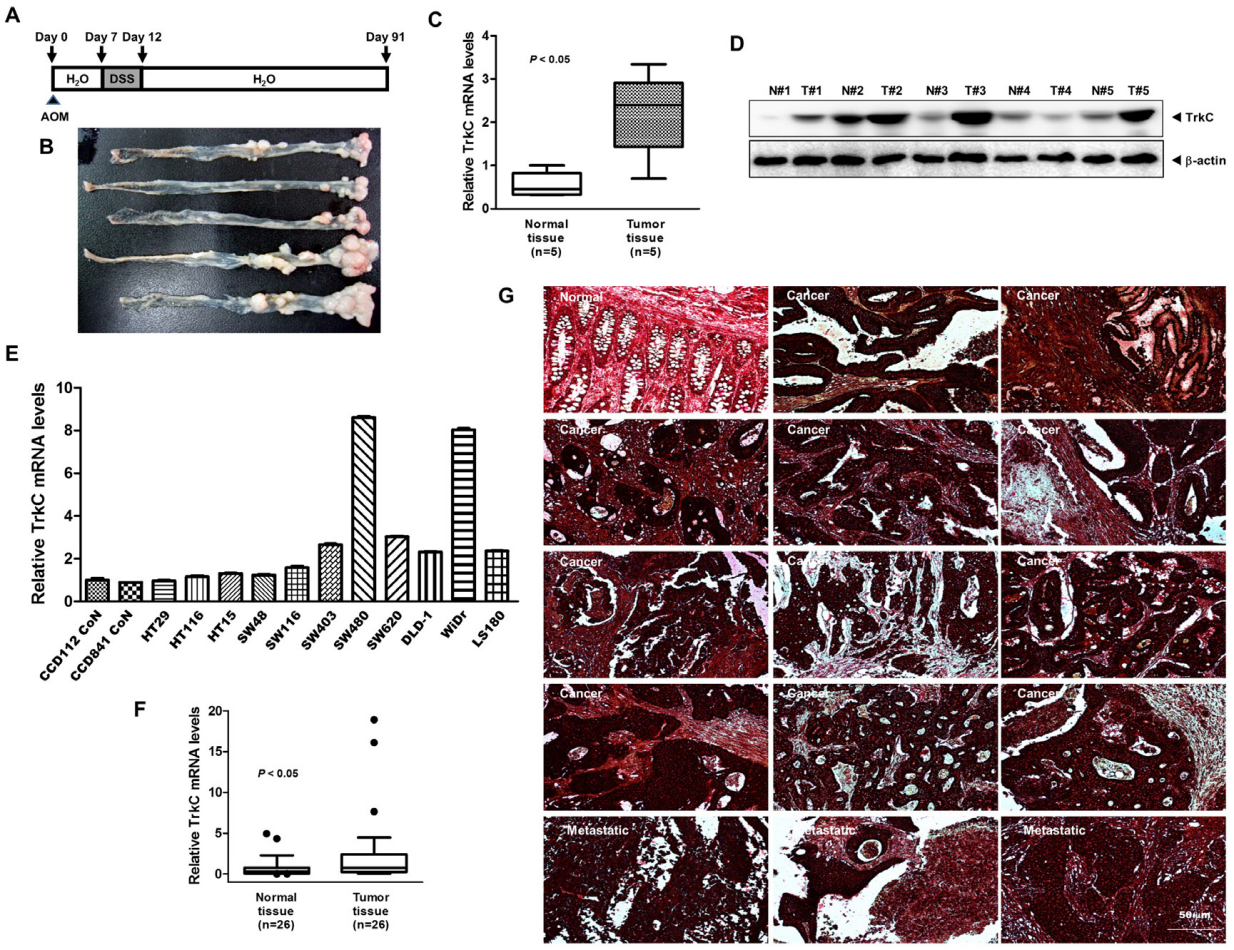
Expression patterns of TrkC in human CRC cells and samples **(A)** Schematic overview of the AOM/DSS CRC model. **(B)** Representative image of colon adenocarcinomas formation on day 91 of AOM/DSS-induced colon cancer. **(C)** The relative levels of TrkC expression in the distal colon from five AOM/DSS-treated and five control mice were assessed by TaqMan real-time quantitative PCR analysis. The endogenous 18S mRNA level was measured as the internal control. The Student's t-test was performed to assess statistical significance (*P < 0.05). **(D)** Western blot analysis of TrkC expression in the distal colon from five AOM/DSS-treated and five control mice. **(E)** Expression of TrkC mRNA in a panel of human normal colon (CCD841 CoN and CCD112 CoN) or CRC cells was examined by TaqMan real-time quantitative PCR analysis. The endogenous 18S mRNA level was measured as the internal control. Error bars represent the mean ± SD of triplicate experiments. *P* < 0.05 was considered to indicate significance in ANOVA. **(F)** The relative levels of TrkC expression of individual human 26 normal or 26 CRC samples were assessed by TaqMan real-time quantitative PCR analysis. Expression was compared with that in healthy tissue. The endogenous 18S mRNA level was measured as the internal control. The Student's t-test was performed to assess statistical significance (*P < 0.05). **(G)** Immunohistochemical analysis of TrkC protein levels in human colon normal, colon adenocarcinoma and metastatic colon adenocarcinoma in lymph nodes. E-cadherin was measured as the epithelial maker.

To determine whether TrkC is involved in CRC, we used an azoxymethane (AOM)/dextran sulphate sodium (DSS) model, in which a single systemic injection of AOM induces colon tumorigenesis in mice induced by chronic DSS administration. AOM/DSS-induced tumors occur preferentially at the distal part of the colon, which is the predominant localization of spontaneous CRC in humans [20]. After AOM/DSS treatment (Figure 2A), all mice developed well-differentiated adenocarcinomas in the distal colon (Figure 2B). We next examined TrkC and NT-3 expression in tumors of AOM/DSS-treated mice. In the AOM/DSS-induced CRC model, the TrkC mRNA and protein levels was significantly higher in AOM/DSS-induced colon tumor tissues than in normal areas of colon in the same mice (Figure 2C and 2D). Also, the expression level of NT-3 mRNA in tumor tissues was higher than normal tissues in AOM/DSS-induced CRC model (Supplementary Figure 3A). Moreover, there was no change in cell motility of SW480 and WiDr control-shRNA or TrkC-shRNA cells after treatment with NT-3 (Supplementary Figure 3B and Supplementary Figure 3C). These observations led us to investigate the expression status of TrkC in CRC cells. TrkC was highly expressed in invasive and metastatic human CRC cells, with the exception of HT29 and HCT116 cells, relative to normal colon cells (CCD112 CoN and CCD841 CoN) (Figure 2E). To further determine whether TrkC plays a role in colon cancer, we next examined the expression of TrkC in 26 tumor samples accompanied by patient-matched samples of normal colon tissues. Interestingly, TrkC expression was elevated in 23 out of 26 tumors (88.5%) relative to the corresponding patient-matched normal tissue samples (Figure 2F). In addition, TrkC expression was more strongly upregulated in CRC tissues than in normal tissues of CRC (Supplementary Figure 4).

Based on these observations, we evaluated TrkC expression using immunohistochemistry in a series of normal and CRC samples. We found a significant correlation between TrkC overexpression and pathological phenotypes. Normal colon tissue samples demonstrated weak immunoreactivity to an anti-TrkC antibody; however, the TrkC level was elevated in CRCs. Moreover, the TrkC level was high in metastatic CRCs of lymph nodes (Figure 2G). Our observation contrasts starkly with a previous study, which demonstrated that CRC cells and tissues contained low TrkC levels than normal tissues [12], and our results raise the possibility that NT-3-independent activation of TrkC via overexpression regulates tumor progression and survival in CRC.

### TrkC is required for the tumorigenic and metastatic ability of CRC

**Figure 3:**
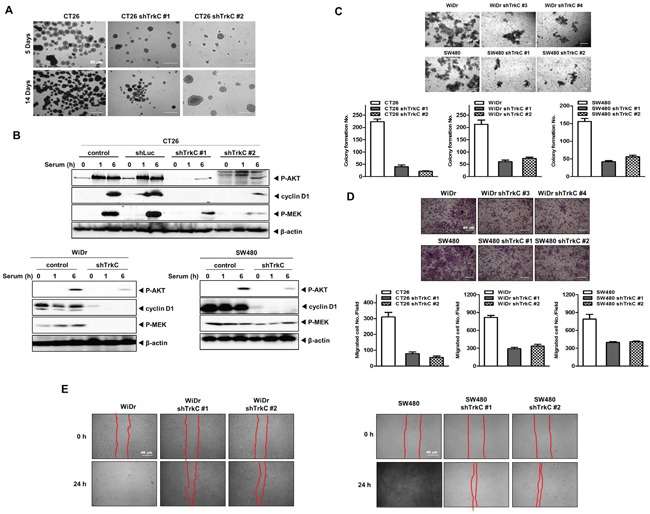
Contribution of TrkC to the metastatic ability of CRC **(A)** CT26 cells infected with the indicated shRNAs grown for 7 days in ultra-low cluster plates and visualized with bright-field microscopy. **(B)** Western blot analysis of expression of phospho-AKT, phospho-MEK1/2 and cyclin D1 in CT26, WiDr and SW480 cells infected with the indicated shRNAs. β-actin was used as a loading control. **(C)** Colony-forming assay of CT26, WiDr and SW480 cells infected with the indicated shRNAs (n = 3). The Student's t-test was performed to assess statistical significance (*P* < 0.05). **(D)** Migration assay of control-shRNA- or shTrkC-treated CT26, WiDr and SW480 cells. Cells that migrated to the bottom of the chamber were counted in five fields (n = 3). The Student's t-test was performed to assess statistical significance (*P* < 0.05). **(E)** Wound healing assay of WiDr and SW480 cells infected with the indicated shRNAs. Wound closures were imaged at 0, 12 and 24 h after wounding.

We speculated that TrkC may be functionally linked to the metastatic potential of CRC. To test this notion, we selected highly metastatic mouse or human CRC cells (CT26, WiDr and SW480) stably expressing control-shRNA or TrkC-targeting shRNA (shTrkC) to suppress its expression. To achieve distant dissemination, cancer cells must overcome anoikis by the formation of large cellular aggregates in suspension to allow cancer cell survival during systemic circulation, thereby facilitating secondary tumor formation [21]. In addition, epithelial-mesenchymal transition (EMT) is involved in anoikis resistance in colon cancer cells [22]. We test whether TrkC could influence the ability of CT26 cells to survive and proliferate. CT26 cells proliferated as large spheroid aggregates in suspension, whereas CT26 TrkC-shRNA cells demonstrated significantly lower survival in suspension (Figure 3A).

The PI3K/AKT and Ras/MAPK pathways are one of the major signalling cascades in CRCs, and aberrant activation of these pathways contributes to poor outcomes, tumor development and EMT induction in CRC [23, 24]. These observations led us to speculate that TrkC might modulate the PI3K/AKT and Ras/MAPK pathways in tumorigenicity and metastasis of CRCs. We examined activation of MEK1/2 and PI3K/AKT by TrkC. Relative to RIE-1 cells expressing pLNCX-TrkC [14], and CT26, WiDr and SW480 cells, control RIE-1 cells, or CT26, WiDr, SW480 TrkC-shRNA cells had significantly reduced levels of phosphorylated (activated) MEK1/2 and AKT as well as cyclin D1 (Figure 3B and Supplementary Figure 5A). Moreover, CT26 cells, WiDr cells, SW480 cells, and RIE-1-TrkC cells were 2.5 ~ 5 fold enriched in colony-forming cells relative to CT26, WiDr, and SW480 TrkC-shRNA cells, and control RIE-1 cells (Figure 3C and Supplementary Figure 5B). Furthermore, WiDr or SW480 TrkC-shRNA cells significantly increased the activation of caspase-3 and cleavage of PARP relative to control-shRNA cells (Supplementary Figure 7). These findings suggest that TrkC affects survival in CRC via induction of cell signalling, leading to the blocking of apoptosis.

To further investigate other functional hallmarks of cancer, we conducted *in vitro* motility and wound healing assays. RIE-1-TrkC cells, CCD112 Con-TrkC, CT26, WiDr and SW480 cells had increased motility relative to control RIE-1 cells, CCD112 Con, or CT26, WiDr, and SW480 TrkC-shRNA cells (Figure 3D, 3E, Supplementary Figure 5C, Supplementary Figure 5D, and Supplementary Figure 5E). Also, CCD112 CoN-TrkC cell had increased motility relative to control CCD112 CoN cells but there was no change in TrkC-induced cell motility after treatment with NT-3 (Supplementary Figure 6A).

More generally, a variety of cell-surface receptors that are configured much like the EGFR receptor have been found in human tumors to be overexpressed and autophosphorylation by their overexpression is linked to marked aggressiveness and poor prognosis [25, 26]. These results suggest that TrkC, in addition to activation of TrkC by binding of NT-3, might has a high capacity for ligand-independent activation by receptor overexpression, even in the absence of bound ligand [5, 7, 27].

We next examined whether the pharmacological inhibition of TrkC with LOXO-101 could influence the ability of CCD112 CoN-TrkC cells to cell motility. LOXO-101, a highly selective inhibitor of Trk tyrosine kinase, showed dramatic clinical activity in patients with a variety of cancers in phase I trials and is undergoing multicenter phase II trials [28]. Cell motility by LOXO-101 treatment was dramatically inhibited relative to that of control (Supplementary Figure 6B). These results demonstrate that tyrosine kinase activity of TrkC is required for metastatic potential of CRC. These various data indicate that TrkC is necessary for the induction of the metastatic potential of CRC, in contrast to inhibition of tumorigenicity by TrkC overexpression previously reported [12, 13].

### TrkC controls tumorigenicity and metastasis of CRC

**Figure 4:**
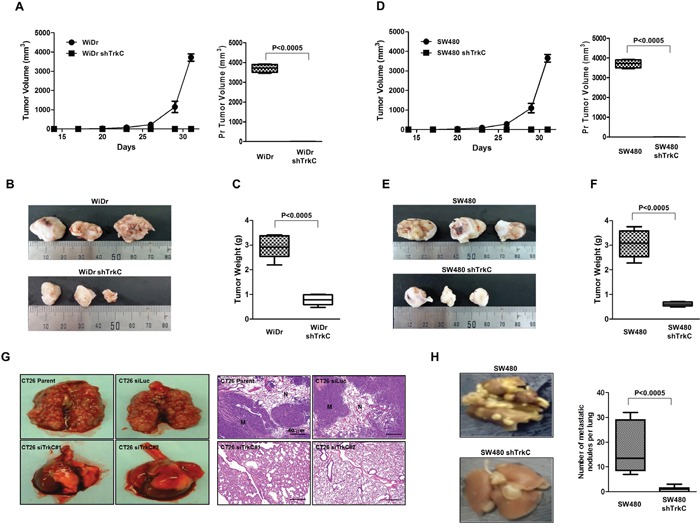
Suppression of TrkC expression inhibits tumorigenicity and metastasis of CRCs **(A)** Tumor formation by WiDr cells infected with the indicated shRNAs. In total, 1.0 × 10^5^ cells were implanted into the mammary fat pads of mice (n = 7). The Student's t-test was performed to assess statistical significance (*P* < 0.0005). **(B)** Representative images of tumors from mice harboring WiDr cells infected with the indicated shRNAs. **(C)** Tumor weights from mice harbouring WiDr cells expressing control-shRNA or TrkC-shRNA. (n = 7). The Student's t-test was performed to assess statistical significance (*P* < 0.0005). **(D)** Tumor formation by SW480 cells infected with the indicated shRNAs. In total, 1.0 × 10^5^ cells were implanted into the mammary fat pads of mice (n = 7). The Student's t-test was performed to assess statistical significance (*P* < 0.0005). **(E)** Representative images of tumors from mice harbouring SW480 cells infected with the indicated shRNAs. **(F)** Tumor weights from mice harbouring SW480 cells infected with the indicated shRNAs (n = 7). The Student's t-test was performed to assess statistical significance (*P* < 0.0005). **(G)** Representative images of lungs and immunohistochemical images of haematoxylin and eosin staining in sections of lungs from individual mice harbouring CT26 cells infected with the indicated shRNAs. **(H)** Representative images and total number of lung metastatic nodules in each mouse in each group after tail vein injection of SW480 control-shRNA or TrkC-shRNA cells (n = 7). The Student's t-test was performed to assess statistical significance (*P* < 0.0005).

To test whether TrkC knockdown alters the tumorigenic and metastatic behaviours of WiDr and SW480 cells, we injected these cells expressing TrkC-shRNA or control-shRNA into the mammary fat pads of BALB/c Nu/Nu mice and examined the resulting primary tumors 31 days later. Implantation of WiDr cells was necessary for subcutaneous tumor formation, whereas primary tumor formation was markedly decreased upon implantation of shTrkC-expressing WiDr cells (Figure 4A). Primary tumors originating from WiDr cells were almost 5-fold heavier on average than those originating from WiDr TrkC-shRNA cells (Figure 4B and 4C). Similar to the observed behaviour of WiDr cells, SW480 cells had an increased primary tumor formation ability. Mice injected with SW480 cells formed large tumors, while very small tumors arose when an equal number of shTrkC-expressing cells was injected (Figure 4D). Moreover, tumors originating from SW480 cells were almost 4-fold heavier than those originating from SW480 TrkC-shRNA cells (Figure 4E and 4F). These results suggest that TrkC promotes their ability to proliferate and survive *in vivo*.

In addition, similar to our published results [14], histological analyses of lung sections of mice injected with CT26 control-shRNA or TrkC-shRNA cells via the tail vein confirmed that the number of micrometastatic lesions was drastically reduced in lungs of mice with CT26 TrkC-shRNA cell-derived tumors relative to control (Figure 4G). Moreover, the average number of visible metastatic nodules was markedly reduced in mice injected with SW480 TrkC-shRNA cells relative to that of mice harboring SW480 control-shRNA cells (Figure 4H). Furthermore, the presence of small numbers of nodules in the lungs of mice carrying SW480 TrkC-shRNA cells was due to knockdown of TrkC relative to control (Supplementary Figure 8A). These results indicate that loss of TrkC reduces both the number of metastases in the lung and cell motility, in contrast to suppression of tumor formation by TrkC overexpression in a xenograft mouse model previously reported [12, 13], and these results indicate that TrkC plays a role in one or more steps of the metastatic process, which makes colonisation of distant organs more efficient.

### TrkC is required for acquisition of self-renewal traits of CRCs by induction of EMT

**Figure 5:**
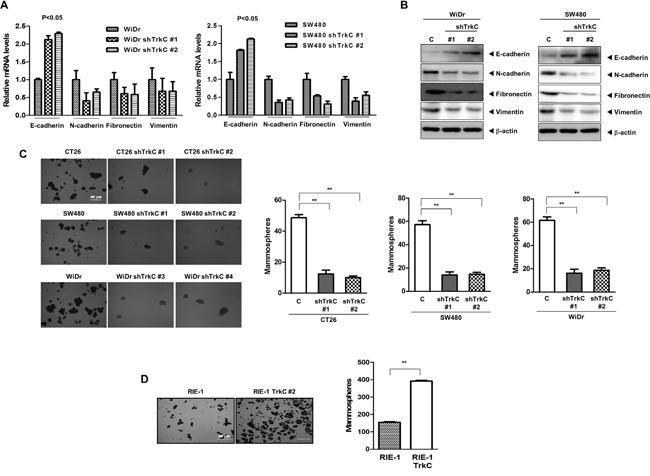
TrkC as a key regulator of the EMT program and maintenance of the CSC state **(A)** mRNA expression levels of E-cadherin, N-cadherin, fibronectin and vimentin in WiDr and SW480 cells infected with the indicated shRNAs. 18S mRNA was used to normalise variability in template loading. The Student's t-test was performed to assess statistical significance (*P* < 0.05). **(B)** Western blot analysis of the expression of E-cadherin, N-cadherin, fibronectin and vimentin proteins in SW480 cells infected with the indicated shRNAs. β-actin was used as a loading control. **(C)** Tumor spheroids formation assay of CT26, WiDr and SW480 cells infected with the indicated shRNAs. Cells were counted in five fields (n = 3). The Student's t-test was performed to assess statistical significance (*P < 0.001). **(D)** Tumor spheroids formation assay of control or RIE-1-TrkC cells. Cells were counted in five fields (n = 3). The Student's t-test was performed to assess statistical significance (*P < 0.001).

During tumor metastasis, cancer cells undergoing EMT express mesenchymal components and manifest a migratory phenotype [29, 30]. Moreover, the EMT program is associated with the tumorigenicity, metastasis, and the self-renewal capability of CRC [31–33]. These observations led us to speculate that the contribution of TrkC to tumorigenicity and metastasis of CRC involves induction of the EMT program. To address this possibility, we examined whether TrkC was able to promote the EMT program. We found that TrkC knockdown had significantly downregulated expression of mesenchymal markers such as N-cadherin, fibronectin and vimentin, but upregulated expression of epithelial markers such as E-cadherin (Figure 5A and 5B). Also, the expression of E-cadherin was markedly induced but significantly reduced expression of mesenchymal markers (N-cadherin, fibronectin and vimentin) in lung of mice injected with SW480 TrkC-shRNA cells relative to that of mice harboring SW480 control-shRNA cells (Supplementary Figure 8B). We next examined the expression levels of EMT-TFs, such as Foxc2, SIP1, Slug, Snail, Twist-1, and Twist-2 in lung of mice injected with SW480 control-shRNA or TrkC-shRNA cells. The expression of EMT-TFs in the lungs of mice with SW480 TrkC-shRNA cells was drastically lower than that of SW480 control-shRNA cells (Supplementary Figure 8C). These results provide further evidence that TrkC contributes to the tumorigenicity and metastasis of CRC.

Based on these observations, our attention was drawn to the possibility that TrkC may affect acquirement of the self-renewal trait associated with CSCs. Tumor spheroids are enriched in early progenitor/stem cells and CSCs [34, 35]. In addition, an ability to form larger and more tumor spheroids promotes mammary stem cell self-renewal and proliferation *in vitro* and increases ductal/alveolar development in humanised NOD-SCID mammary fat pads [36, 37]. To test this notion, we tested the tumor spheroids-forming abilities by TrkC to gauge the stem cell content of these cultures. CT26, WiDr and SW480 cells were 3.9-fold, 3.4-fold and 4-fold enriched in tumor spheroids-forming cells, respectively, relative to the corresponding shTrkC-expressing cells (Figure 5C). Furthermore, RIE-1-TrkC cells formed 2.5-fold more tumor spheroids than RIE-1 cells infected with the control vector (Figure 5D). In addition, the expression of stem cell markers in the lungs of mice with SW480 TrkC-shRNA cells was markedly reduced relative to that of SW480 control-shRNA cells (Supplementary Figure 8D). These results suggesting that TrkC itself induces the self-renewal of mammary stem cell through induction of EMT. Our findings are in contrast to another study of colon cancer, which demonstrated that TrkC does not affect the EMT program or transforming growth factor (TGF)-β signalling [12].

### TrkC is essential for primary tumor formation and metastasis of CRC via inhibition of TGF-β signalling

Several lines of evidence indicate that TGF-β plays an important role as a tumor suppressor during colorectal carcinogenesis by inhibiting cell proliferation and inducing apoptosis [38–40]. We therefore explored whether TrkC modulates TGF-β signaling to activate tumorigenicity in CRC. we examined TGF-β1 transcriptional activity using a TGF-β-responsive SBE4- and 3TP-luc reporter construct in RIE-1 and CT26 cells to determine whether TrkC expression has any effect on TGF-β signalling. TGF-β1-induced SBE4 and 3TP transcriptional activity was significantly reduced in CT26 cells than in RIE-1 cells (Supplementary Figure 9A and Supplementary Figure 9B). Furthermore, TGF-β1-mediated SBE4- and 3TP-luciferase activity was efficiently suppressed in SW480 and WiDr cells compared with normal colon cells (CCD841 Con) (Supplementary Figure 9C and Supplementary Figure 9D). These results correlated with Smad2 and Smad3 phosphorylation, an indicator of active TGF-β signalling. TGF-β1 significantly stimulated endogenous Smad2/3 phosphorylation in RIE-1 and CCD841 Con cells. However, the level of TGF-β1-stimulated Smad2/3 phosphorylation was markedly lower in CT26, WiDr and SW480 cells than in RIE-1 and CCD841 Con cells (Supplementary Figure 9E and Supplementary Figure 9F).

Moreover, inhibition of Trk kinase activity by K252a [41] inhibited growth of CT26 cells compared with RIE-1 cells (Supplementary Figure 9G). We then examined whether inhibition of TrkC signalling enhances TGF-β1-induced inhibition of DNA synthesis by measuring thymidine incorporation. CT26 cells were resistant to TGF-β1-induced growth inhibitory activity. However, inhibition of Trk kinase activity by K252a restored TGF-β1-induced growth inhibitory activity in CT26 cells (Supplementary Figure 9H). These findings indicate that TrkC kinase activity is required for suppression of TGF-β signalling.

We assessed the effects of TrkC expression on TGF-β1-mediated SBE4- or 3TP-luciferase activity in RIE-1 cells. Transient transfection of TrkC suppressed TGF-β1-induced transcriptional activity in RIE-1 cells (Supplementary Figure 10A and Supplementary Figure 10B). we next examined Smad2/3 phosphorylation and growth inhibition by TGF-β1. TGF-β1-mediated Smad2/3 phosphorylation was markedly reduced and TGF-β1-induced growth inhibitory activity was significantly repressed in RIE-1-TrkC cells (Supplementary Figure 10C and Supplementary Figure 10D) compared with control cells. Consistent with this suppressive activity of TrkC, expression of TrkC dramatically repressed the TGF-β1-dependent activity of the SBE4-luc and 3TP-luc reporters (Supplementary Figure 10E and Supplementary Figure 10F).

**Figure 6:**
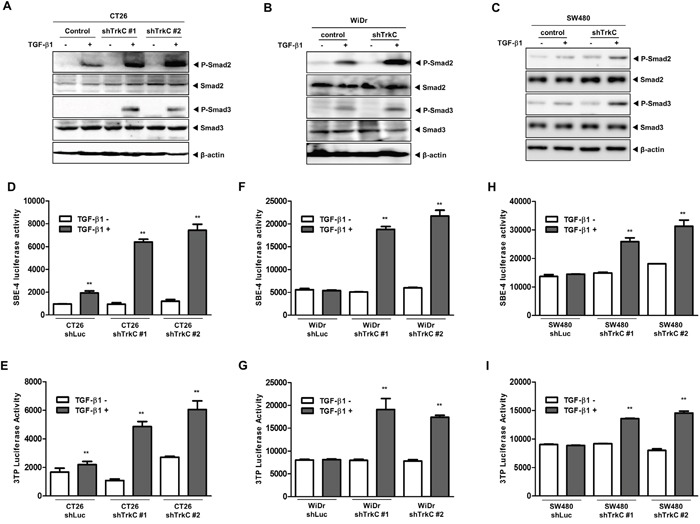
Knockdown of TrkC restores TGF-β signalling **(A)** Western blot analysis of the expression of phospho-Smad2, phospho-Smad3, Smad2 and Smad3 proteins in CT26 cells infected with the indicated shRNAs after stimulation with TGF-β1 (5 ng/mL). β-actin was used as a loading control. **(B)** Western blot analysis of the expression of phospho-Smad2, phospho-Smad3, Smad2 and Smad3 proteins in WiDr cells infected with the indicated shRNAs after stimulation with TGF-β1 (5 ng/mL). β-actin was used as a loading control. **(C)** Western blot analysis of the expression of phospho-Smad2, phospho-Smad3, Smad2 and Smad3 proteins in SW480 cells infected with the indicated shRNAs after stimulation with TGF-β1 (5 ng/mL). β-actin was used as a loading control. **(D, E)** Luciferase reporter assay of TGF-β1-responsive SBE **(D)** or 3TP **(E)** in CT26 cells infected with the indicated shRNAs. Luciferase activity was measured 24 h after treatment with TGF-β1. **Control versus treatment with TGF-β1, *P* < 0.001. n = 3. **(F, G)** Luciferase reporter assay of TGF-β1-responsive SBE **(F)** or 3TP **(G)** in WiDr cells infected with the indicated shRNAs. Luciferase activity was measured 24 h after treatment with TGF-β1. **Control versus treatment with TGF-β1, *P* < 0.001. n = 3. **(H, I)** Luciferase reporter assay of TGF-β1-responsive SBE **(H)** or 3TP **(I)** in SW480 cells infected with the indicated shRNAs. Luciferase activity was measured 24 h after treatment with TGF-β1. *Control versus treatment with TGF-β1, *P* < 0.001. n = 3.

These observations led us to investigate the activation status of Smad2/3 in CT26, WiDr and SW480 cells expressing control-shRNA or TrkC-shRNA. Activation of Smad2/3 was markedly higher in CT26, WiDr, and SW480 TrkC-shRNA cells than in the corresponding control-shRNA cells (Figure 6A, 6B and 6C). This suggests that TGF-β signalling underlies the inhibition of proliferation in CT26 TrkC-shRNA cells (Supplementary Figure 11A). Moreover, knockdown of TrkC significantly enhanced TGF-β1-induced SBE4- and 3TP-luciferase activity, relative to CT26, WiDr and SW480 cells (Figure 6D–6I), which correlated with increased activity of a Smad-reporter plasmid (Supplementary Figure 11B). TGF-β1-mediated cell growth inhibition occurs through induction of cell-cycle-arrest-related genes, such as *PAI-1*, *p15^Ink4b^* and *p21^Waf1/Cip1^* [42, 43]. To verify a function of TrkC as a negative regulator of TGF-β signaling *in vivo*, we next examined the expression of TGF-β1 target genes such as *p15^Ink4b^*, *p21^Waf1/Cip1^*, and PAI1 by RT-PCR in SW480 and WiDr control-shRNA or TrkC-shRNA cells. These results demonstrated that TrkC overexpressing cells responded with diminished sensitivity to TGF-β1 in terms of induction of TGF-β1 target genes (Supplementary Figure 11C). These results suggest that TrkC expression attenuates TGF-β1 tumor suppressor activity.

### TrkC blocks TGF-β type II receptor (TβRII)/TGF-β type I receptor (TβRI) complex formation via inactivation and downregulation of TβRII

**Figure 7:**
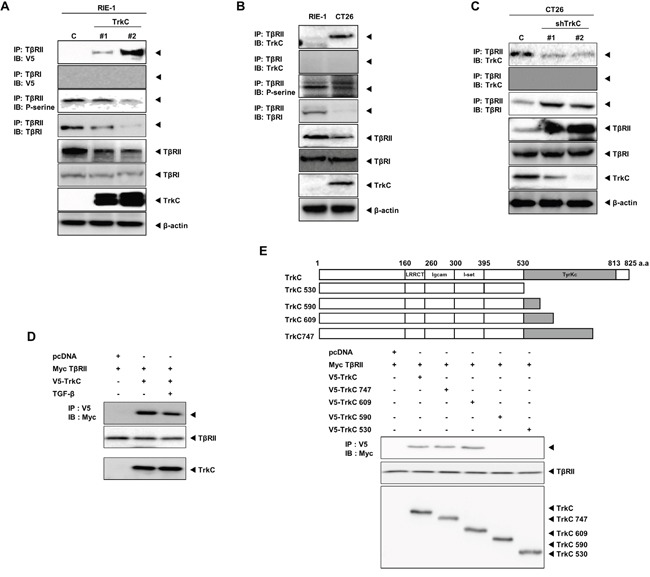
TrkC blocks TβRII/TβRI complex formation via TrkC/TβRII interaction **(A)** Identification of TrkC/TβRII complexes in control and RIE-1-TrkC cells. Cell lysates were subjected to immunoprecipitation using an anti-TβRII antibody followed by immunoblotting with the indicated antibodies. β-actin was used as a loading control. **(B)** Identification of endogenous TrkC/TβRII complexes in RIE-1 and CT26 cells. Cell lysates were subjected to immunoprecipitation using anti-IgG and anti-TβRII antibodies followed by immunoblotting with the indicated antibodies. β-actin was used as a loading control. **(C)** Identification of endogenous TrkC/TβRII complexes in CT26 cells infected with the indicated shRNAs. Cell lysates were subjected to immunoprecipitation using an anti-TβRII antibody followed by immunoblotting with the indicated antibodies. β-actin was used as a loading control. **(D)** Identification of TrkC/TβRII complexes with or without TGF-β1. Immunoblot analysis of whole-cell lysates and immunoprecipitates derived from 293T cells transfected with V5-TrkC and Myc-TβRII constructs as indicated. **(E)** Immunoblot analysis of whole-cell lysates and immunoprecipitates derived from 293T cells transfected with V5-TrkC deletion constructs and Myc-TβRII constructs as indicated.

Our results suggest that the actions of TrkC are positioned upstream of Smad2 and Smad3 phosphorylation; therefore, we examined the possibility that TrkC directly interacts with TβRII in normal colon cells and colon cancer cells. TrkC interacted strongly with TβRII, but not with TβRI (Figure 7A). Furthermore, serine phosphorylation and the expression level of TβRII were markedly reduced in RIE-1-TrkC cells compared with the control. Moreover, the level of TβRI bound to TβRII was markedly reduced in the presence of TrkC.

We next examined the endogenous interaction between TrkC and TβRII in CT26 cells, which express TrkC, and in RIE-1 cells, which do not express TrkC. Endogenous TrkC strongly interacted with TβRII, but did not interact with TβRI. The phosphorylation and expression level of TβRII were reduced in CT26 cells. Moreover, endogenous TβRII/TβRI complex formation was significantly reduced in CT26 cells (Figure 7B). However, TrkC knockdown markedly increased the level of TβRII, and greatly enhanced endogenous TβRII/TβRI complex formation (Figure 7C). we also examined whether TrkC-TβRII interaction depends on TGF-β. TrkC strongly interacted with TβRII with or without TGF-β (Figure 7D). These results suggest that TrkC interacted strongly with TβRII in a ligand-independent manner.

In a previous report, sequence analysis of tyrosine in CRCs revealed four point mutations within the *trkC* kinase-encoding domain. The effect of these *trkC* mutations on kinase function remains to be determined; however, their positions in the sequence suggest that many of them are activating [44]. To assess the effects of point mutants of TrkC on TGF-β signalling, we examined SBE4- or 3TP-luciferase activity by TrkC mutants and wild-type TrkC in RIE-1 cells. TrkC point mutants and wild-type TrkC suppressed TGF-β1-induced transcriptional activity in RIE-1 cells. However, luciferase activity revealed no obvious difference in suppression of TGF-β signalling between wild-type TrkC and point mutants of TrkC (Supplementary Figure 12). These findings strongly indicate that TrkC inhibits TGF-β signalling through its interaction with TβRII and that this association inhibits TβRII/TβRI complex formation.

To identify the functional domain of TrkC responsible for the interaction with TβRII, we used a series of deletion constructs of TrkC. The TrkC 609, TrkC 747 and TrkC 825 deletion mutants interacted with TβRII, whereas the TrkC 530 and TrkC 590 deletion mutants did not. These results suggest that TβRII interacts with residues 591–609 of the tyrosine kinase domain of TrkC (Figure 7E).

Previous reports suggested that TGF-β induced EMT and invasion, and that TGF-β inhibitor decreased EMT and metastatic potential in CT26 and SW480 cells [45–47]. To investigate whether TrkC knockdown could influence TGF-β induced EMT, we assessed the expression of epithelial and mesenchymal markers. We found that TGF-β treatment induced mesenchymal markers such as N-cadherin, fibronectin and vimentin, but upregulated expression of epithelial markers such as E-cadherin in SW480 and WiDr control-shRNA cells. However, in SW480 and WiDr TrkC-shRNA cells, TGF-β treatment reduced expression of mesenchymal markers such as N-cadherin, fibronectin and vimentin (Supplementary Figure 13). These results provide further evidence that TrkC is essential for TGF-β induced EMT program and also contributes to the tumorigenicity and metastasis of CRC.

## DISCUSSION

Although TrkC plays an important role in the initiation and progression of CRC, two recent studies proposed that TrkC is a tumor suppressor as a dependence receptor in CRC. Previous paper has reported that TrkC have a tumor progressive function and may be metastatic marker in CRC. TrkC knockdown by TrkC siRNA treatment decreased cell survival and invasion. In addition, TrkC knockdown in WiDr and Colo320 cells suppressed the expression of TGF-beta, AKT1, MMP2/9, and mTOR. However, the signaling mechanisms that induce tumorigenicity and metastasis of CRC by TrkC have remained poorly understood. Also, we reported that TrkC directly binds to the bone morphogenetic protein type II receptor and inhibits bone morphogenetic protein signaling in CRC [14]. However, another two studies proposed that TrkC acts as a tumor suppressor in CRC. Upregulation of NT-3 and TrkC expression in normal colon tissues relative to CRC tissues and CRC cells was correlated with NT-3 and TrkC methylation. Also, TrkC induces apoptosis in colorectal cancers in absence of NT-3. Moreover, the loss of TrkC expression associates with neoplastic transformation of CRC [12, 13]. Although their findings are interesting and well-presented, these reports suggest that the role of TrkC in CRC is controversial and led us to investigate whether TrkC is a tumor suppressor.

Surprisingly, we found that TrkC expression was markedly elevated in CRC tissues of patients relative to normal tissue samples, correlating with increased expression of NT-3, through analysis of TrkC and NT-3 expression using publicly available microarray, RNA-sequence and patient clinical data. Furthermore, TrkC was strongly correlated with the recurrence, pathogenesis and survival of CRC patients; however, NT-3 expression did not significantly differ to CRC stages or patient survival. Moreover, TrkC was highly expressed in invasive and metastatic human CRC cells and tissues of CRC patients relative to normal colon. These observations suggest that TrkC acts as an activator of CRC carcinogenesis.

We further assessed the effects of TrkC on the acquisition of the metastatic ability of CRC. TrkC significantly induces cell migration and invasion. Moreover, TrkC led to the constitutive activation of two major effector pathways, the Ras/MAPK mitogenic pathway and the PI3K/AKT pathway, and induction of cyclin D1 mediating cell survival. Furthermore, our observations indicate that TrkC itself induces the self-renewal ability of mammary stem cell through induction of EMT. These results suggest that the increased tumor spheroids formation ability by TrkC forces the generation of stem cell-like cells through induction of the self-renewal properties of CRC cells, consistent with previous reports that tumor spheroids are enriched in early progenitor/stem cells and CSCs [34, 35]. Furthermore, our observations provide direct support for the results of other, which demonstrated that cortical progenitor cells express TrkC to activate progenitor cell survival via activation of the PI3K/AKT and Ras/MEK pathways [48]. In addition, in an *in vivo* model of experimental tumor growth and metastasis, TrkC augmented metastatic colonisation during the progression of CRC by promoting extravasation, cell survival and migration within the microenvironment of the lung. These observations indicate that tumor growth and metastasis of CRC depend on TrkC expression. Thus, TrkC in CRC does not seem to fit as a tumor suppressor via its function as previously reported.

Current study show that TrkC does not affect the EMT program and TrkC-induced apoptosis is increased in CRCs [12]. However, in this study, we demonstrated that TrkC plays a critical role in EMT and maintenance of the mesenchymal/self-renewal traits of CRCs. Further support of this biological concept comes from the work of others, demonstrating that CSCs are associated with EMT-mediated invasion of CRC [31, 32] and the PI3K/AKT pathway was preferentially activated in CSCs by induction of EMT [49]. Moreover, an increase in phospho-ERK2 by ERK2 upregulation induced EMT in CRC [31]. This finding indicates that TrkC increases CSC traits through activation of the PI3K/AKT pathway and induction of EMT.

One important signalling pathway in CRC is the TGF-β signalling pathway. This pathway serves as a tumor suppressor pathway in the CRCs by inhibiting cell proliferation and inducing apoptosis [40]. Also, up to 74% of colon cancer cell lines have become resistant to the anti-proliferative effects of TGF-β [50], and TβRII serves as a tumor suppressor in CRC. Mice that lack TβRII in the colon epithelium developed more colorectal adenomas and adenocarcinomas and exhibited higher neoplastic proliferation than mice with intact TβRII [51]. These studies suggest that TβRII inactivation contributes to the transformation of CRCs. By contrast, previous study demonstrated TGF-β and bone morphogenetic protein signalling was unaffected by TrkC expression [12]. We therefore explored the contributions of TrkC to activation of CRC pathogenesis via inhibition of TGF-β signalling. Interestingly, TrkC blocked TGF-β-mediated growth inhibition and markedly reduced TGF-β1-stimulated Smad2/3 phosphorylation. Moreover, TrkC interacted strongly with TβRII, but not with TβRI, to block the ability of TβRII to recruit TβRI by reducing the phosphorylation and expression of TβRII. Furthermore, TrkC is essential for TGF-β-induced EMT and invasion. It is also possible that cross-talk between TrkC receptor signalling and TGF-β signalling is required for CRC progression. In summary, our results indicate that TrkC provides important molecular insights into CRC and that there is a novel functional link between hallmarks of cancer and TrkC (Supplementary Figure 14). Our findings also suggest that TrkC activity and/or expression has a potential use as a therapeutic strategy for CRC.

## MATERIALS AND METHODS

### Cell culture and reagents

Rat intestinal epithelial (RIE-1), CCD841 Con and CCD112 Con (Normal colon epithelial cell), mouse and human colorectal cancer cells, and 293T cell were maintained with complete medium according to American Type Culture Collection (ATCC) recommendations. The protein kinase inhibitor K252a was purchased from Calbiochem.

### Human colorectal tumor samples

The biospecimens for this study were provided by the Ajou University Human Bio-Resource Bank (AHBB), a member of Korea Biobank Network, which is supported by the Ministry of Health and Welfare. All samples were obtained with informed consent under institutional review board-approved protocols. (IRB approval number: AJHB-2013-02, Supplementary Table 1).

### Immunohistochemistry

For human tissues, a tissue microarray slide (CO702b) was purchased from US Biomax. The sections and tissue microarray slide were subjected immunohistochemistry as previously described [52].

### TrkC shRNA screen

pLKO.1 lentiviral plasmids encoding mouse TrkC-shRNAs have been described [14]. For human TrkC knockdown were performed using MISSION shRNA pLKO.1-puro plasmids from Sigma. WiDr, and SW480 cells were infected with the TrkC-shRNA lentivirus and selected for 3 days with 1 mg/ml puromycin. An shRNA that does not match any known mouse or human coding cDNA was used as control.

### Antibodies, immunoblotting, and immunoprecipitation

All assays were performed as previously described [53]. Antibodies were obtained from the following sources: anti-TGF-β type I (H-100), anti- TGF-β type II (C-20), and anti-Myc (9E10) from Santa Cruz; anti-V5 (R960CUS) from Invitrogen; anti-TrkC (43078), anti-phospho-serine(17464), and anti-β-actin (8226) from Abcam; anti-phospho-AKT (4060), anti-cyclin D1 (2922), anti-phospho-Smad2 (3108), anti-phospho-Smad3 (9520), anti-Smad2 (3122), anti-Smad3 (9513), and anti-phospho-MEK1/2 (9154) from Cell Signaling Technology; anti-E-cadherin (610182), anti-fibronectin (563098), anti-N-cadherin (610920), and anti-vimentin (550513) from BD Biosciences.

### Colony forming assay, anchorage-independent cell growth assay, tumor spheroids assay, wound healing assay, and invasion assay

All assays were performed as previously described [53]. For the migration assay, 10,000 cells were seeded into 24-well culture inserts with 8 μm pores (BD, 353097). For wound healing assay, 100,000 cells were seeded into 6-well culture. For anchorage-independent cell growth and tumor spheroids assays, 10,000 cells were seeded into 6-well ultra-low adhesion plates. The tumor spheroids assay measures anchorage-independent proliferation at clonal density *in vitro* but not Anchorage-independent Cell Growth Assay. For visualization, the cells were fixed and stained with crystal violet. Three fields per filter were counted.

### RNA preparation, RT-PCR, and quantitative RT-PCR analysis

The primer sequences are listed in the supplemental experimental procedures (Supplementary Table 2). RNA Preparation, RT-PCR, and Quantitative RT-PCR Analysis were performed as previously described [53]. Specific human TrkC (Hs00176797_m1), mouse TrkC (Mm00456222_m1), human 18S (Hs99999901_s1), and mouse 18S (01310297_g1) quantitative probes for Taqman RT-PCR were obtained from Applied Biosystems.

### Luciferase reporter assay

Cells that were 50% confluent in 12-well dishes were transfected using Lipofectamine 2000 (Invitrogen). Luciferase reporter assay were performed as previously described [53]. All of the experiments were performed in triplicate.

### In silico analysis of clinical microarray data

In silico analysis of the published clinical microarray or RNA-sequence data was performed using the GSE20916 [14, 15], GSE28722 [16], and TCGA datasets [17, 18]. *TrkC* and *NT-3* gene expression signatures in the datasets from colorectal cancer patients were extracted and averaged. Graphs with gene expression and survival analysis were plotted using GraphPad Prism v 5.0 (GraphPad Software, Inc.).

### Mouse models of colon cancer and xenograft implantation of trkc-shRNA cells

Female BALB/c Nu/Nu mice (7 weeks old, *n* = 7), and C57BL/6 mice (7 weeks old, *n* = 5) were handled in compliance with protocols approved by the Institutional Animal Care and Use Committee (IACUC) of Gachon University (Approval No. LCDI-2013-0029). For the colitis-associated colon cancer model, we followed a published protocol [20, 54]. The C57BL/6 mice were injected a single intraperitoneal injection with 10 mg/kg of azoxymethane (AOM; Sigma) or PBS. 7 days later, mice received 2% DSS or drinking water for 5 days. For animal studies, we performed as previously described [5]. For tumorigenicity studies, 1 × 10^5^ cells suspended in 50μl PBS/Matrigel (BD Biosciences) were injected subcutaneously into the left and right hind flank regions under anesthesia. Mice were euthanized at 5 weeks, and primary tumors were excised for analysis. For tail-vein injection, 1 × 10^5^ cells suspended in 50μl PBS were injected into the tail vein of 7-week-old Female BALB/c Nu/Nu mice.

### Statistical analysis

Data are expressed as the means ± SEM. Statistical analyses of the data were conducted via Student's t test (two-tailed) and ANOVA. Differences were considered statistically significant at *P* < 0.05 or *P* < 0.0001.

## SUPPLEMENTARY MATERIALS FIGURES AND TABLES






