# Supplementary material for: TrkC promotes colorectal cancer growth and metastasis
Source: Oncotarget. 2017 Apr 20;8(25):41319–33. doi: 10.18632/oncotarget.17289 (PMC5522271; doi:10.18632/oncotarget.17289)
Supplement: Supplementary file 1 [file oncotarget-08-41319-s001.pdf]

TrkC promotes colorectal cancer growth and metastasis

SUPPLEMENTARY FIGURES AND TABLES

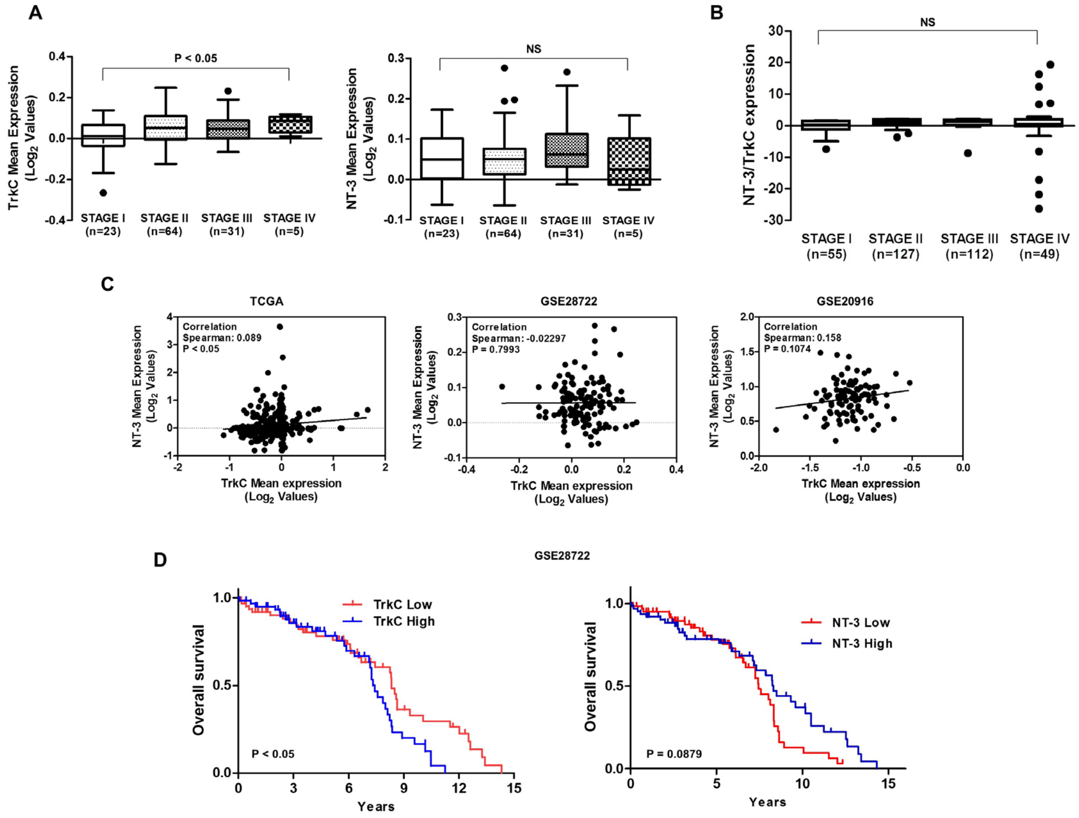

Supplementary Figure 1: TrkC expression is correlated with the survival status of CRC patients. (A) Mean expression of TrkC and NT-3 obtained through analysis of the GSE28722 dataset was plotted as box plots according to tumor stages. TrkC and NT-3 levels were extracted from the dataset and averaged in each tumor. Points below and above the whiskers are drawn as individual dots. $P < 0.05$ was considered to indicate significance in ANOVA. NS, not significant. **(B)** NT-3/TrkC expression obtained through RNA-sequence analysis of 629 CRC patients in the TCGA dataset was plotted as box plots. TrkC and NT-3 levels were extracted from the dataset and averaged in each tumor. Points below and above the whiskers are drawn as individual dots. $P < 0.05$ was considered to indicate significance in ANOVA. NS, not significant. **(C)** Indirect correlation between NT-3 expression and TrkC expression. Scatter plots showing the correlation of TrkC expression with NT-3 expression in the TCGA, GSE28722, and GSE20916 datasets. The *r* value was calculated via Spearman's rank correlation coefficient analysis. **(D)** CRC patients from the GSE28722 dataset were divided into high and low TrkC expressers, and overall survival was compared. *P* values correspond to the log-rank test comparing the survival curves.

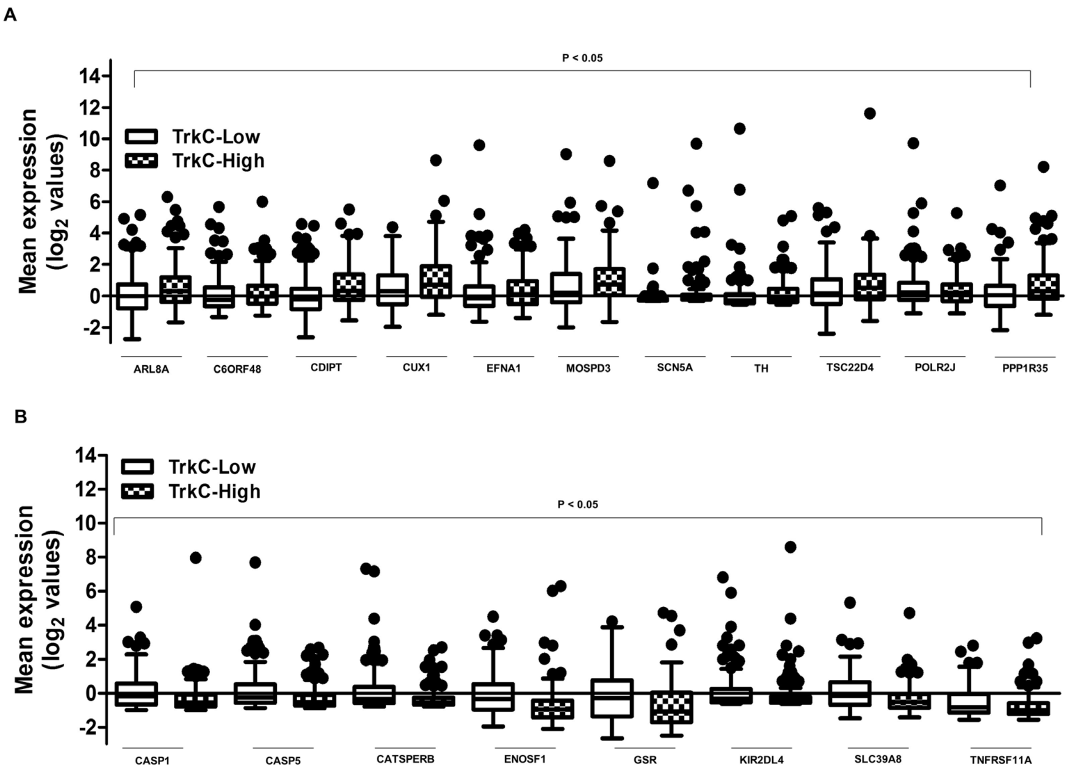

Supplementary Figure 2: TrkC expression is correlated with expression of markers associated with aggressive and less-aggressive tumors. (A) In total, 629 CRC patients from the TCGA dataset were divided into high and low TrkC expressers, and expression of molecular markers associated with tumor aggressiveness was compared. The Student's t-test was performed to assess statistical significance ($P < 0.05$). **(B)** In total, 629 CRC patients from the TCGA dataset were divided into high and low TrkC expressers, and expression of molecular markers associated with less-aggressive tumor was compared. The Student's t-test was performed to assess statistical significance ($P < 0.05$).

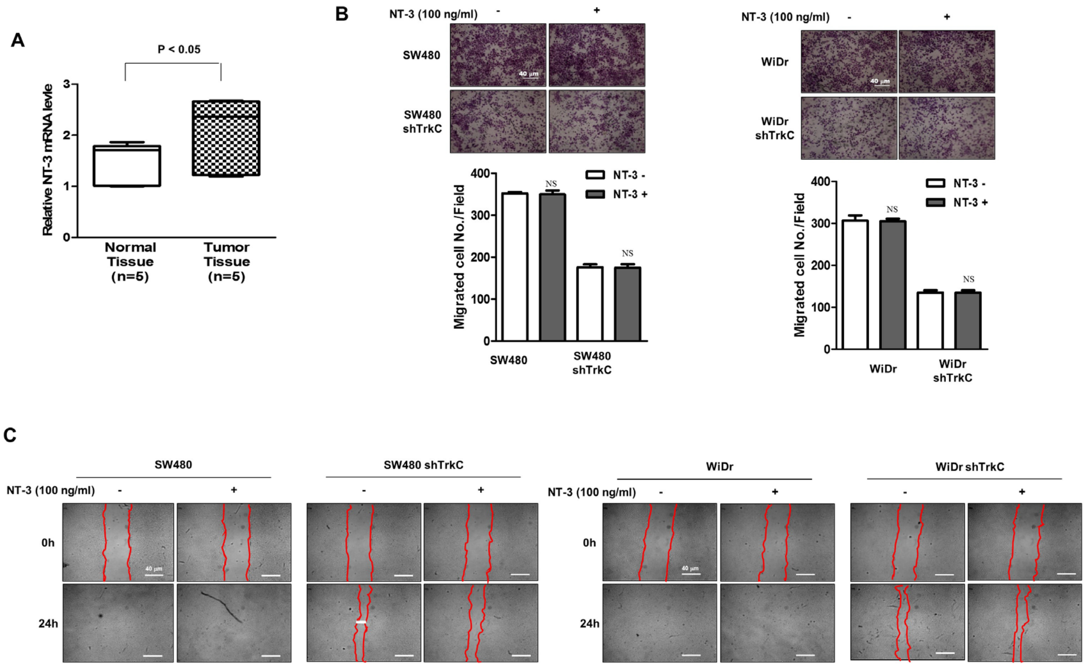

Supplementary Figure 3: Expression patterns of NT-3 in AOM/DSS CRC model and the effect of NT-3 in TrkC-induced metastatic potential of CRC. **(A)** The relative levels of NT-3 expression in the distal colon from five AOM/DSS-treated and five control mice were assessed by quantitative real-time PCR analysis. The endogenous 18S mRNA level was measured as the internal control. The Student's t-test was performed to assess statistical significance ($P < 0.05$). **(B)** Migration assay of SW480 and WiDr control-shRNA or TrkC-shRNA cells with or without 100 ng/ml NT-3 treatment (n=3). *t*-test, NS; not significant. The Student's t-test was performed to assess statistical significance ($P < 0.05$). **(C)** Wound healing assay of SW480 and WiDr control-shRNA or TrkC-shRNA cells with or without 100 ng/ml NT-3 treatment. Wound closures were photographed at 0 and 24 hrs after wounding.

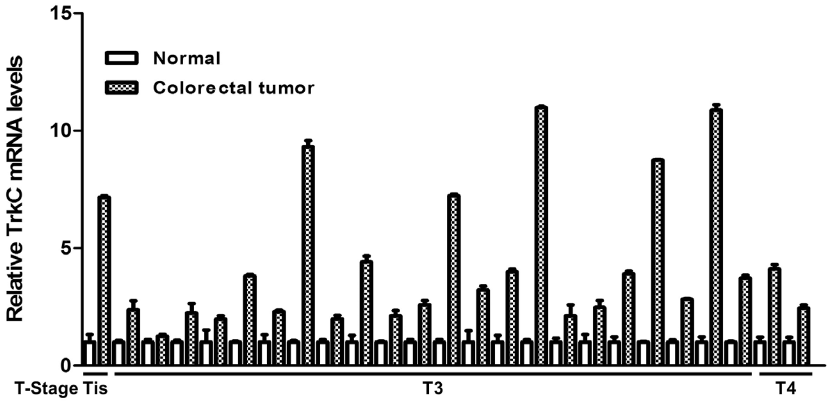

Supplementary Figure 4: Expression patterns of TrkC in stages of 26 human CRC patients. The relative levels of TrkC expression in stages of individual human 26 normal or 26 CRC samples were assessed by TaqMan real-time quantitative PCR analysis. Expression was compared with that in healthy tissue. The endogenous 18S mRNA level was measured as the internal control. The Student's t-test was performed to assess statistical significance ($P < 0.001$).

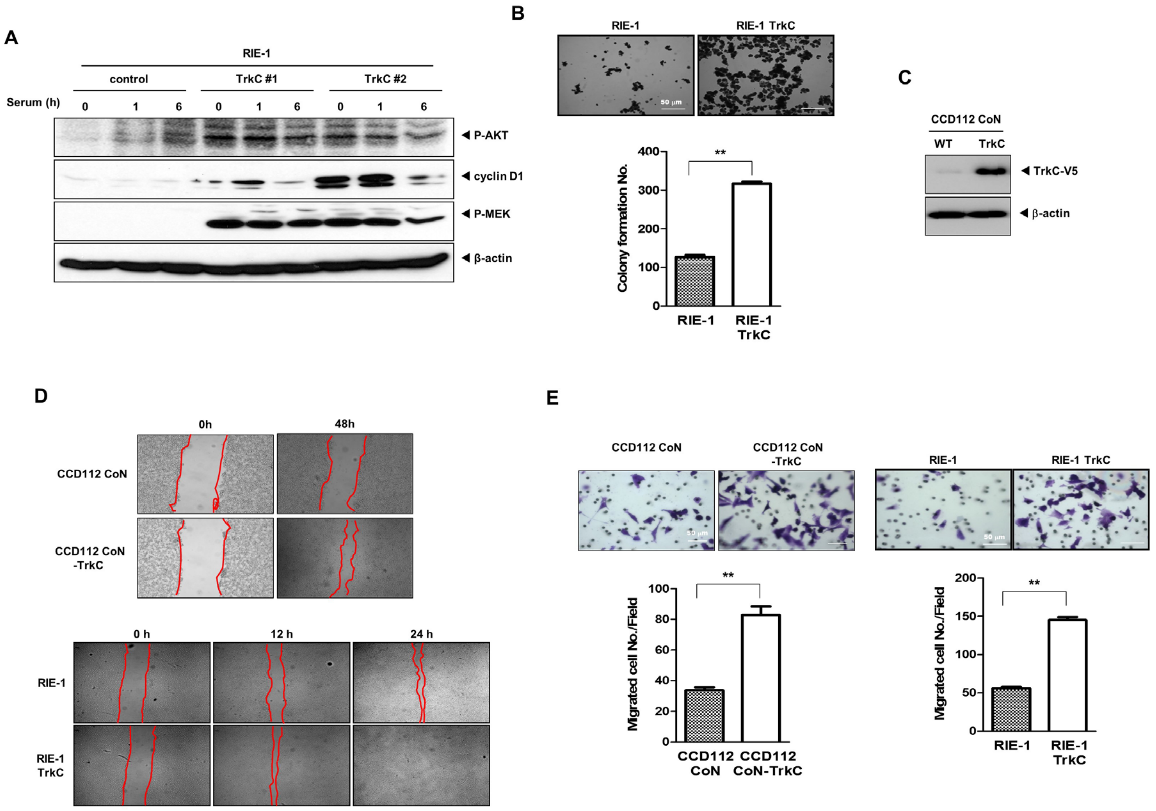

Supplementary Figure 5: TrkC induces the metastatic ability of RIE-1 and CCD112 CoN cells. **(A)** Western blot analysis of expression of phospho-AKT, phospho-MEK1/2 and cyclin D1 in RIE-1 cells infected with pLNCX or pLNCX-TrkC constructs. β-actin was used as a loading control. **(B)** Soft agar colony-forming assay of RIE-1 cells infected with pLNCX or pLNCX-TrkC constructs. Monolayer cells were trypsinised, washed and plated in medium containing 0.2% agar to assess anchorage-independent growth. Results are presented as the number of macroscopic colonies formed at 3 weeks after plating. The Student's t-test was performed to assess statistical significance (**$P < 0.001$). **(C)** Western blot analysis of the expression of TrkC in CCD112 CoN control or TrkC cells. β-actin was used as a loading control. **(D)** Wound healing assay of RIE-1 and CCD112 CoN cells infected with pLNCX or pLNCX-TrkC constructs. Wound closures were imaged at 0, 12, 24, and 48 h after wounding. **(E)** Quantification of the migratory abilities of RIE-1 and CCD112 CoN cells infected with pLNCX or pLNCX-TrkC constructs by a migration assay. Assays were performed in triplicate, and the averages with SEM are shown. The Student's t-test was performed to assess statistical significance (**$P < 0.001$).

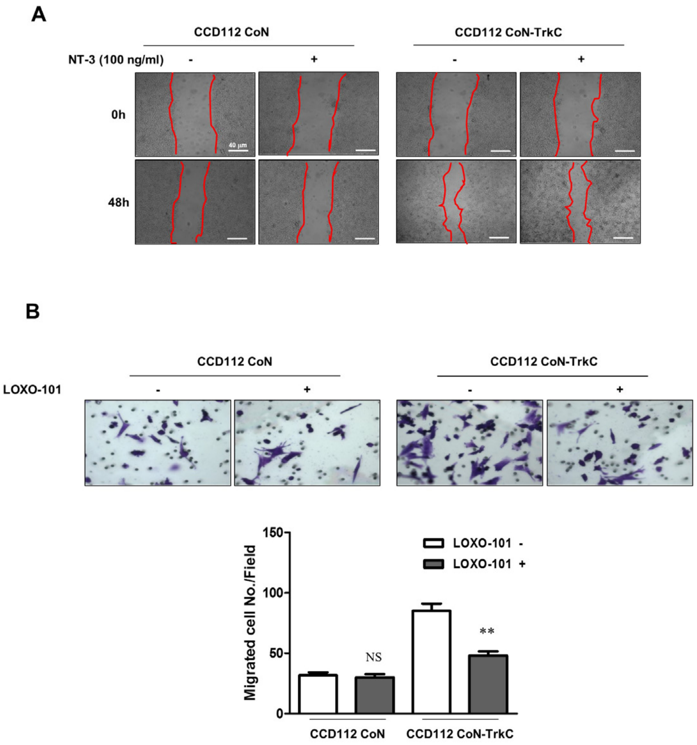

Supplementary Figure 6: The effect of NT-3 and LOXO-101 in TrkC-induced metastatic potential of CRC. **(A)** Wound healing assay of CCD112 CoN or CCD112 CoN-TrkC cells with or without 100 ng/ml NT-3 treatment. Wound closures were photographed at 0 and 48 hrs after wounding. **(B)** Migration assay of CCD112 CoN or CCD112 CoN-TrkC cells with or without 5μM LOXO-101 treatment (n=3). $^{**}P < 0.001$, *t*-test, NS; not significant.

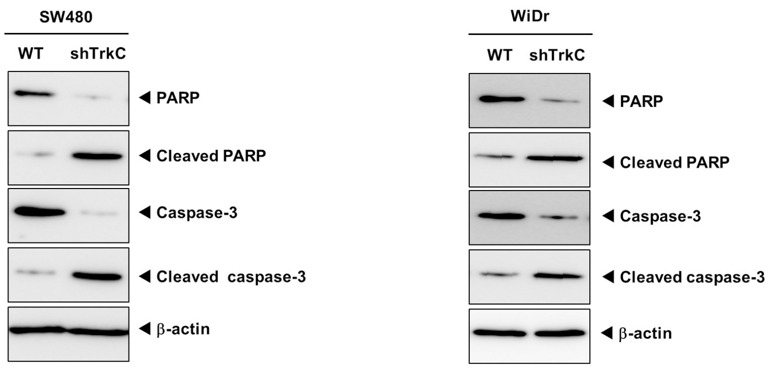

Supplementary Figure 7: TrkC inhibits apoptosis of CRC cells. Cleavage of procaspase-3 and PARP of WiDr, SW480 control-shRNA or TrkC-shRNA cells was analyzed by Western blotting.

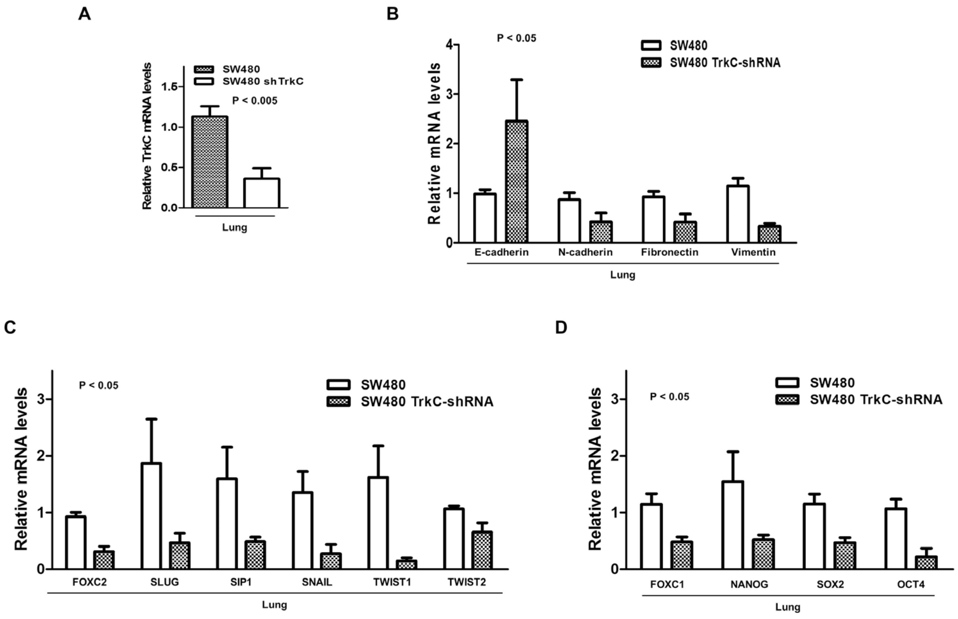

Supplementary Figure 8: Contribution of TrkC to the metastatic ability of SW480 cells. (A) Quantitative RT-PCR of the expression of TrkC in the lungs of individual mice expressing either SW480 control-shRNA or TrkC-shRNA. Data are presented as mean ± standard error of the mean (SEM), n = 3. The Student's t-test was performed to assess statistical significance ($P < 0.005$). **(B)** Quantitative RT-PCR of the expression of E-cadherin, N-cadherin, fibronectin, and vimentin in the lungs of individual mice expressing either SW480 control-shRNA or TrkC-shRNA. Data are presented as mean ± standard error of the mean (SEM), n = 3. The Student's t-test was performed to assess statistical significance ($P < 0.05$). **(C)** Quantitative RT-PCR of the expression of EMT-TFs such as FOXC2, SLUG, SIP-1, SNAIL, TWIST-1, and TWIST-2 in the lungs of individual mice expressing either SW480 control-shRNA or TrkC-shRNA. Data are presented as mean ± standard error of the mean (SEM), n = 3. The Student's t-test was performed to assess statistical significance ($P < 0.05$). **(D)** Quantitative RT-PCR of the expression of stem cell makers (FOXC1, NANOG, SOX2, and OCT4) in the lungs of individual mice expressing either SW480 control-shRNA or TrkC-shRNA. Data are presented as mean ± standard error of the mean (SEM), n = 3. The Student's t-test was performed to assess statistical significance ($P < 0.05$).

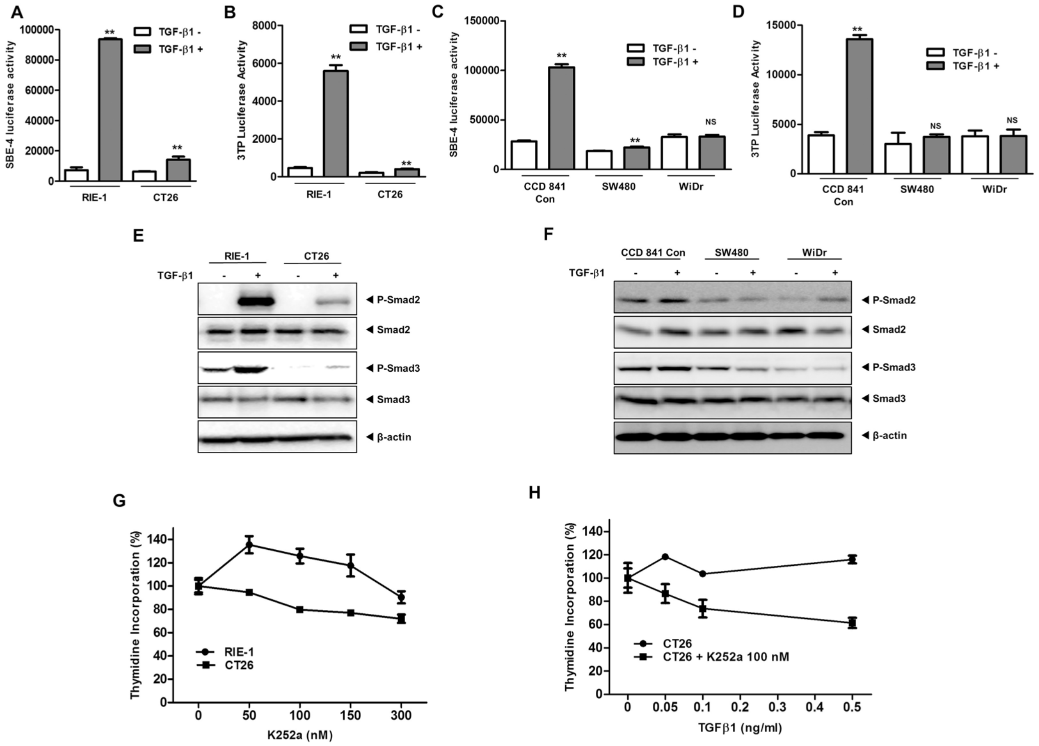

Supplementary Figure 9: Restriction of TGF-β signalling in highly metastatic CRC cells. (A, B) Luciferase reporter assay of TGF-β1-responsive SBE **(A)** or 3TP **(B)** in RIE-1 and CT26 cells. Luciferase activity was measured 24 h after treatment with TGF-β1. **Control versus treatment with TGF-β1, $P < 0.001$. n = 3. **(C, D)** Luciferase reporter assay of TGF-β1-responsive SBE **(C)** or 3TP **(D)** in CCD841 Con, SW480 and WiDr cells. Luciferase activity was measured 24 h after treatment with TGF-β1. **Control versus treatment with TGF-β1, $P < 0.001$. n = 3. NS; not significant. **(E)** Western blot analysis of the expression of phospho-Smad2, phospho-Smad3, Smad2 and Smad3 proteins in RIE and CT26 cells after stimulation with TGF-β1 (5 ng/mL). β-actin was used as a loading control. **(F)** Western blot analysis of the expression of phospho-Smad2, phospho-Smad3, Smad2 and Smad3 proteins in CCD841 Con, SW480 and WiDr cells after stimulation with TGF-β1 (5 ng/mL). β-actin was used as a loading control. **(G)** Thymidine incorporation assay of RIE-1 and CT26 cells treated with various concentrations of K252a as indicated. Points, averages of means from three determinations; bars, SD. **(H)** Thymidine incorporation assay of RIE-1 and CT26 cells treated with various concentrations of TGF-β1 with or without K252a (100 nM) as indicated. Points, averages of means from three determinations; bars, SD.

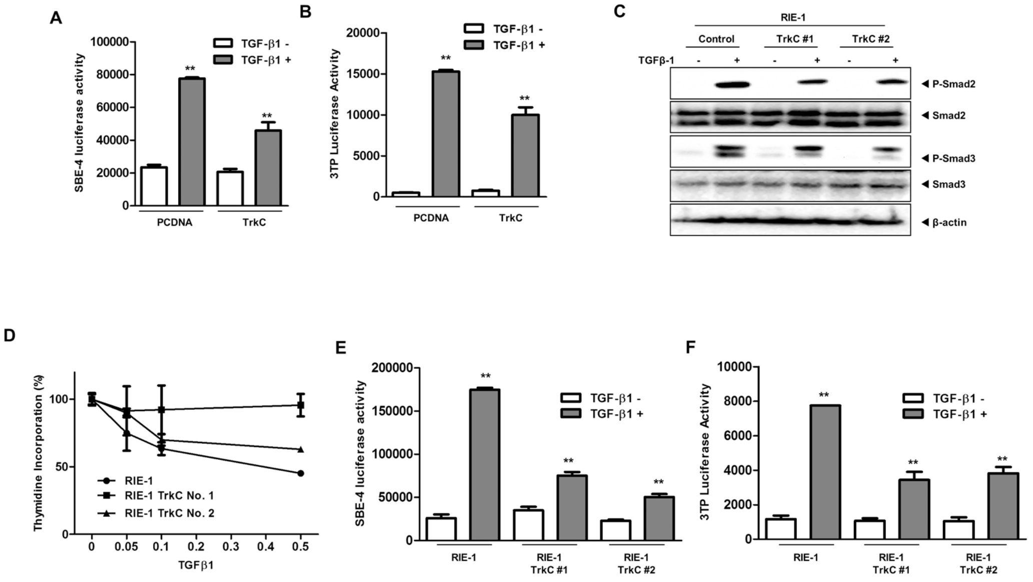

Supplementary Figure 10: TrkC overexpression inhibits TGF-β signalling. (A, B) Luciferase reporter assay of TGF-β1-responsive SBE **(A)** or 3TP **(B)** in RIE-1 cells transfected with pCDNA or pCDNA-TrkC expression constructs. Luciferase activity was measured 24 h after treatment with TGF-β1. **Control versus treatment with TGF-β1, $P < 0.001$. n = 3. **(C)** Western blot analysis of the expression of phospho-Smad2, phospho-Smad3, Smad2 and Smad3 proteins in control and RIE-1-TrkC cells after stimulation with TGF-β1 (5 ng/mL). β-actin was used as a loading control. **(D)** Thymidine incorporation assay in control and RIE-1-TrkC cells treated with various concentrations of TGF-β1 as indicated. Points, averages of means from three determinations; bars, SD. **(E, F)** Luciferase reporter assay of TGF-β1-responsive SBE **(E)** or 3TP **(F)** in control and RIE-1-TrkC cells. Luciferase activity was measured 24 h after treatment with TGF-β1. **Control versus treatment with TGF-β1, $P < 0.001$. n = 3.

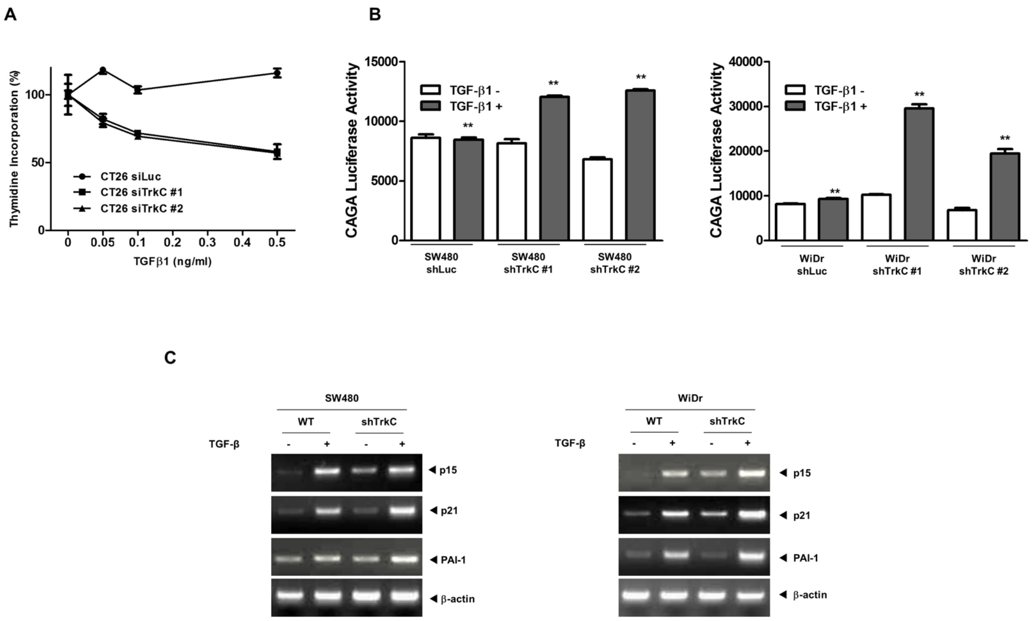

Supplementary Figure 11: TrkC knockdown enhances TGF-β signalling. **(A)** Thymidine incorporation assay of CT26 cells infected and treated as indicated. Points, averages of means from three determinations; bars, SD. **(B)** Luciferase reporter assay of TGF-β1-responsive CAGA in SW480 and WiDr cells infected with indicated shRNAs. Luciferase activity was measured 24 h after treatment with TGF-β1 (5 ng/mL). **Control versus treatment with TGF-β1, $P < 0.001$. n = 3. **(C)** SW480 and WiDr control-shRNA or TrkC-shRNA cells were treated with TGF-β1 for 8hr, and then total RNA was isolated. mRNA levels of TGF-β1 target genes were detected by RT-PCR.

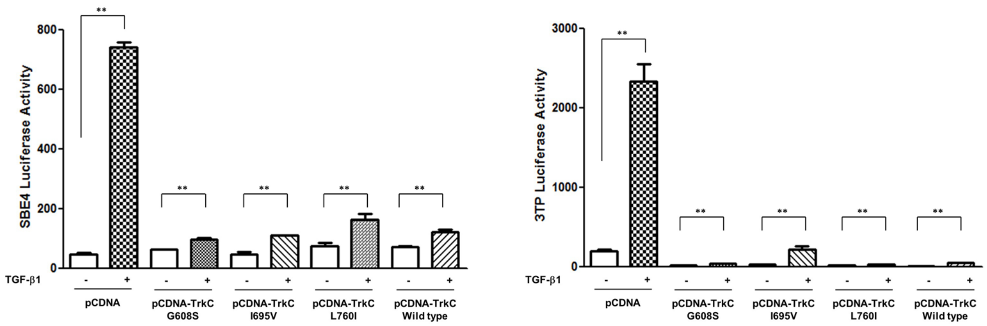

Supplementary Figure 12: TrkC point mutants and wild-type inhibits TGF-β signalling. Luciferase reporter assay of TGF-β1-responsive SBE or 3TP in RIE-1 cells transfected with pCDNA or pCDNA-TrkC, and TrkC point mutants expression constructs. Luciferase activity was measured 24 h after treatment with TGF-β1. **Control versus treatment with TGF-β1, $P < 0.001$. n = 3.

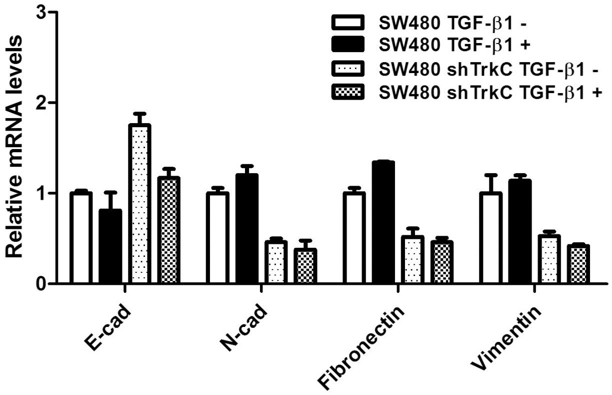

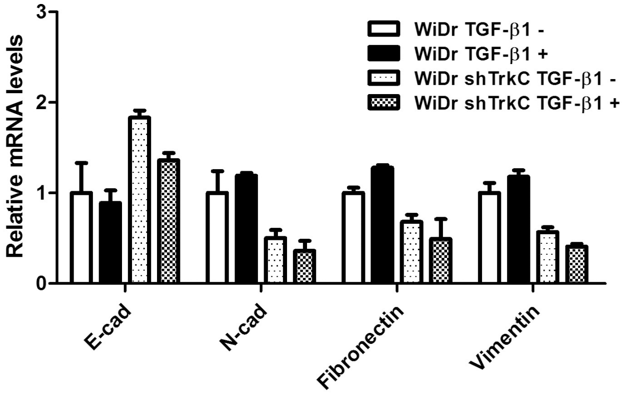

Supplementary Figure 13: The relationship between TrkC and TGF-β1 in induction of EMT program. **(A)** mRNA expression levels of E-cadherin, N-cadherin, fibronectin and vimentin in WiDr and SW480 cells infected with the indicated shRNAs. 18S mRNA was used to normalise variability in template loading. The expression level was measured 36 h after treatment with TGF-β1 (5 ng/mL). The Student's t-test was performed to assess statistical significance ($P < 0.05$). **(B)** Western blot analysis of the expression of E-cadherin, N-cadherin, fibronectin and vimentin proteins in WiDr cells infected with the indicated shRNAs. β-actin was used as a loading control. The expression level was measured 36 h after treatment with TGF-β1 (5 ng/mL). The Student's t-test was performed to assess statistical significance ($P < 0.05$).

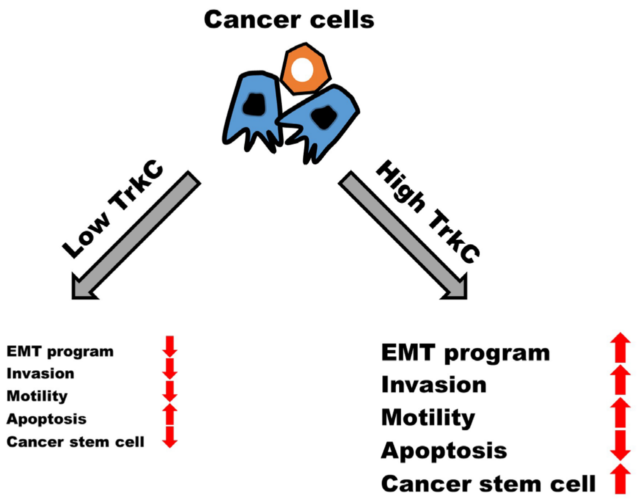

Supplementary Figure 14: The regulation of tumor microenvironment TrkC in CRCs. TrkC induced hallmarks of cancer such as sustaining proliferative signalling and resisting cell death via constitutive activation of Mek1 and Akt, as well as constitutively high expression of cyclin D1 by TrkC. Also, TrkC induced cellular traits associated with motility, invasiveness, malignancy, self-renewal traits, and drug resistance by activation of EMT. Moreover, TrkC blocked TGF-β-mediated growth inhibition.

Supplementary Table 1: Clinicopathological characteristics of normal colon and CRC samples

See Supplementary File 1

Supplementary Table 2: Primer sequences for RT-PCR and quantitative RT-PCR

See Supplementary File 2
