# Supplementary material for: TrkC promotes colorectal cancer growth and metastasis
Source: Oncotarget. 2017 Apr 20;8(25):41319–33. doi: 10.18632/oncotarget.17289 (PMC5522271; doi:10.18632/oncotarget.17289)
Supplement: Supplementary file 2 [file oncotarget-08-41319-s002.doc]

**Table S**1. Clinicopathological characteristics of normal colon and CRC samples

| **patient ID** | **Gender** | **Age** | **Tumor Stage** | **Lymph Node Stage** | **Metastasis Stage** | **Recurrence** | **Neoplasm Anatomic Subdivision** | **ICDO code** | **Overall Survival Status** | **Overall Survival (Months)** | **BRAF**  **Mutation** |
| --- | --- | --- | --- | --- | --- | --- | --- | --- | --- | --- | --- |
| 11-05-1 | Female | 40 | T3 | N2 | M0 | no | sigmoid colon | Adenocarcinoma | Living | 47 | ND |
| 11-13-1 | Female | 69 | T3 | N1 | M0 | no | sigmoid colon | Adenocarcinoma | unknown | 26 | ND |
| 11-15-1 | Male | 70 | T3 | N0 | M0 | no | sigmoid colon | Adenocarcinoma | unknown | 41 | ND |
| 11-17-1 | Male | 48 | T3 | N1 | M0 | no | sigmoid colon | Adenocarcinoma | Living | 46 | ND |
| 11-30-1 | Male | 37 | T3 | N2 | M0 | no | ascending colon | Adenocarcinoma | Living | 46 | ND |
| 11-34-1 | Female | 44 | T3 | N0 | M0 | no | sigmoid colon | Adenocarcinoma | Living | 46 | Negative |
| 11-35-1 | Female | 69 | T4 | N1 | M0 | no | ascending colon | Adenocarcinoma | unknown | 3 | ND |
| 11-54-1 | Male | 76 | T3 | N0 | M0 | no | rectosigmoid junction | Adenocarcinoma | unknown | 33 | Negative |
| 11-59-1 | Female | 54 | T3 | N0 | M0 | no | ascending colon | Adenocarcinoma | unknown | 35 | Negative |
| 11-60-1 | Male | 66 | T3 | N0 | M0 | no | sigmoid colon | Mucinous adenocarcinoma | unknown | 33 | ND |
| 12-02-1 | Male | 52 | T3 | N1 | M1 (liver) | no | sigmoid colon | Adenocarcinoma | Living | 55 | Negative |
| 12-06-1 | Female | 72 | T3 | N0 | M0 | no | ascending colon | Adenocarcinoma | unknown | 10 | Negative |
| 12-09-1 | Male | 52 | T3 | N0 | M0 | no | ascending colon | Adenocarcinoma | Living | 64 | Negative |
| 12-14-1 | Male | 57 | T3 | N2 | M0 | no | rectum | Adenocarcinoma | Living | 38 | Negative |
| 12-15-1 | Male | 59 | T3 | N2 | M0 | no | ascending colon | Adenocarcinoma | unknown | 21 | ND |
| 12-19-1 | Male | 59 | T3 | N1 | M1 (liver) | no | ascending colon | Adenocarcinoma | Living | 38 | Negative |
| 12-21-1 | Female | 54 | T3 | N0 | M0 | no | ascending colon | Adenocarcinoma | Living | 38 | Negative |
| 12-35-1 | Male | 43 | T3 | N2 | M0 | no | rectum | Adenocarcinoma | Living | 37 | Negative |
| 12-40-1 | Female | 69 | T3 | N2 | M0 | no | rectum | Adenocarcinoma | Living | 37 | ND |
| 12-42-1 | Female | 73 | T3 | N0 | M0 | no | sigmoid colon | Adenocarcinoma | Living | 37 | ND |
| 12-75-1 | Male | 77 | T4 | N1 | M1 (liver and splenic) | no | sigmoid colon | Adenocarcinoma | unknown | 2 | ND |
| 12-88-1 | Male | 59 | T3 | N0 | M0 | no | rectum | Adenocarcinoma | Living | 32 | ND |
| 13-10-1 | Male | 68 | T3 | N1 | M0 | no | sigmoid colon | Adenocarcinoma | Deceased | 5 | Negative |
| 13-17-1 | Male | 59 | T3 | N0 | M0 | no | sigmoid colon | Adenocarcinoma | Living | 25 | Negative |
| 13-28-1 | Male | 49 | Tis | N0 | M0 | no | cecum | Adenocarcinoma | Living | 25 | ND |
| 13-31-1 | Female | 77 | T3 | N0 | M0 | no | ascending colon | Adenocarcinoma | Living | 25 | ND |

ND: Not determined
