# Supplementary material for: TrkC promotes colorectal cancer growth and metastasis
Source: Oncotarget. 2017 Apr 20;8(25):41319–33. doi: 10.18632/oncotarget.17289 (PMC5522271; doi:10.18632/oncotarget.17289)
Supplement: Supplementary file 3 [file oncotarget-08-41319-s003.doc]

**Table S2. Primer sequences for RT-PCR and quantitative RT-PCR**

| **Quantitative RT-PCR Primers** | | | |
| --- | --- | --- | --- |
| Gene | Primers | Gene | Primers |
| **Human**  **E-cadherin** | F: 5’-**TGCCCAGAAAATGAAAAAGG**-3’  R: 5’-**GTGTATGTGGCAATGCGTTC**-3’ | **Human**  **FOXC2** | F: 5’- GCCTAAGGACCTGGTGAAGC-3’  R: 5’- TTGACGAAGCACTCGTTGAG-3’ |
| **Human**  **N-cadherin** | F: 5’-**ACAGTGGCCACCTACAAAGG**-3’  R: 5’-**CCGAGATGGGGTTGATAATG**-3’ | **Human**  **Snail** | F: 5’- CCTCCCTGTCAGATGAGGAC-3’  R: 5’- CCAGGCTGAGGTATTCCTTG-3’ |
| **Human**  **Fibronectin** | F: 5’-**CAGTGGGAGACCTCGAGAAG**-3’  R: 5’-**TCCCTCGGAACATCAGAAAC**-3’ | **Human**  **Twist-1** | F: 5’- GGAGTCCGCAGTCTTACGAG-3’  R: 5’- TCTGGAGGACCTGGTAGAGG-3’ |
| **Human**  **Vimentin** | F: 5’-**GAGAACTTTGCCGTTGAAGC**-3’  R: 5’-**GCTTCCTGTAGGTGGCAATC**-3’ | **Human**  **SIP1** | F: 5’- TTCCTGGGCTACGACCATAC-3’  R: 5’- TGTGCTCCATCAAGCAATTC-3’ |
| **Human**  **OCT4** | F: 5’- **GGAGGAAGCTGACAACAATGAAA**-3’  R: 5’- **GGCCTGCACGAGGGTTT**-3’ | **Human**  **Slug** | F: 5’- GGGGAGAAGCCTTTTTCTTG-3’  R: 5’- TCCTCATGTTTGTGCAGGAG-3’ |
| **Human**  **SOX2** | F: 5’- **TGCGAGCGCTGCACAT**-3’  R: 5’- **TCATGAGCGTCTTGGTTTTCC**-3’ | **Human**  **Twist-2** | F: 5’- CGACGAGCTGGACTCCAAG-3’  R: 5’-  CCTCCATCCTCCAGACCGA-3’ |
| **Human**  **Nanog** | F: 5’- **ACAACTGGCCGAAGAATAGCA**-3’  R: 5’- **GGTTCCCAGTCGGGTTCAC**-3’ | **Human**  **Slug** | F: 5’- ACGGCATCTACCAGTTCATC-3’  R: 5’- TCCTTCTCCTCCTTGTCCTT-3’ |
| **Human**  **TβRII** | F: 5’- AGCATCACGGCCATCTGTG-3’  R: 5’- TGGCAAACCGTCTCCAGAGT-3’ | Human  18S | F: 5’-ACCGCAGCTAGGAATAATGGA-3’  R: 5’-GCCTCAGTTCCGAAAACCA-3’ |
| **RT-PCR Primers** | | | |
| Gene | Primers | Gene | Primers |
| **Human**  **P21** | F: 5’-GGCTTCATGCCAGCTACTTC-3’  R: 5’-CCCTAGGCTGTGCTCACTTC-3’ | **Human**  **P15** | F: 5’-CGCCCACAACGACTTTATTT-3’  R: 5’-CACCAGGTCCAGTCAAGGAT-3’ |
| **Human**  **PAI-1** | F: 5’-TCTCAGGAAGTCCAGCCACT-3’  R: 5’-ACCCTCTGGCTGGTAGGTTT-3’ | **Human**  **β-actin** | F: 5’-TCACCCACACTGTGCCCATCTACGA-3’  R: 5’-CAGCGGAACCGCTCATTGCCAATGG-3’ |
